# Role of Hydrophobicity at the N-Terminal Region of Aβ42 in Secondary Nucleation

**DOI:** 10.1021/acschemneuro.2c00504

**Published:** 2022-11-21

**Authors:** Dev Thacker, Amanda Willas, Alexander J. Dear, Sara Linse

**Affiliations:** † Department of Biochemistry and Structural Biology, Lund University, Lund22362, Sweden; ‡ Centre for Misfolding Diseases, Department of Chemistry, University of Cambridge, CambridgeCB2 1EW, U.K.

**Keywords:** Alzheimer’s disease, amyloid, secondary nucleation, hydrophobicity

## Abstract

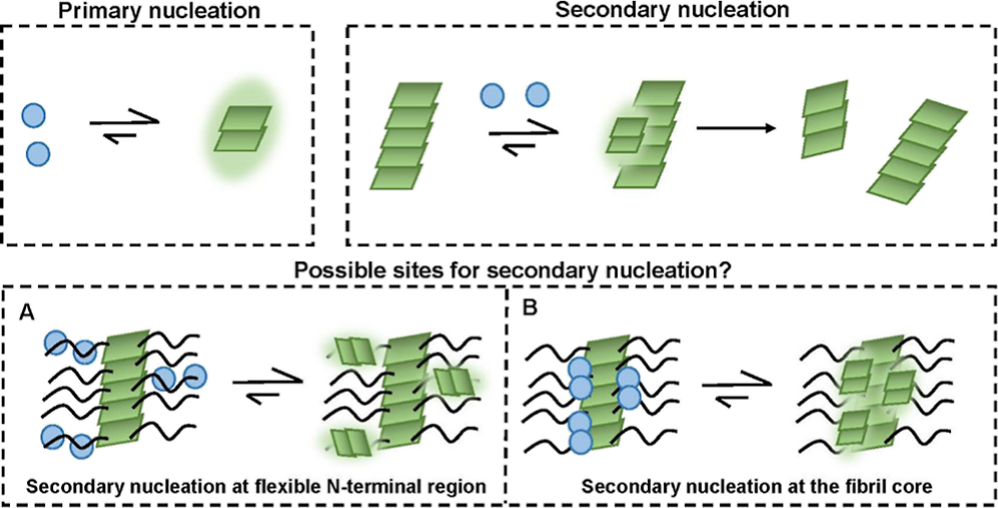

The self-assembly of the amyloid β 42 (Aβ42) peptide is linked to Alzheimer’s disease, and oligomeric intermediates are linked to neuronal cell death during the pathology of the disease. These oligomers are produced prolifically during secondary nucleation, by which the aggregation of monomers is catalyzed on fibril surfaces. Significant progress has been made in understanding the aggregation mechanism of Aβ42; still, a detailed molecular-level understanding of secondary nucleation is lacking. Here, we explore the role of four hydrophobic residues on the unstructured N-terminal region of Aβ42 in secondary nucleation. We create eight mutants with single substitutions at one of the four positions—Ala2, Phe4, Tyr10, and Val12—to decrease the hydrophobicity at respective positions (A2T, A2S, F4A, F4S, Y10A, Y10S, V12A, and V12S) and one mutant (Y10F) to remove the polar nature of Tyr10. Kinetic analyses of aggregation data reveal that the hydrophobicity at the N-terminal region of Aβ42, especially at positions 10 and 12, affects the rate of fibril mass generated via secondary nucleation. Cryo-electron micrographs reveal that most of the mutants with lower hydrophobicity form fibrils that are markedly longer than WT Aβ42, in line with the reduced secondary nucleation rates for these peptides. The dominance of secondary nucleation, however, is still retained in the aggregation mechanism of these mutants because the rate of primary nucleation is even more reduced. This highlights that secondary nucleation is a general phenomenon that is not dependent on any one particular feature of the peptide and is rather robust to sequence perturbations.

## Introduction

Aβ42 is one of the most aggregation-prone variants of the amyloid β peptide involved in the pathology of Alzheimer’s disease (AD).^1−4^ In its native form, Aβ42 is monomeric and unstructured. However, during the pathology of AD, Aβ42 undergoes self-assembly and forms amyloid aggregates through a series of microscopic steps, including primary nucleation, secondary nucleation, and elongation. Primary nucleation involves monomers only, whereas secondary nucleation involves both monomers and already existing aggregates, formed from the same kind of monomers. The aggregates may act as seeds, on the surface of which monomers interact to form nuclei more easily than in solution.^5^ Fibrils thus provide a catalytic surface for nucleation. Secondary nucleation leads to the formation of oligomeric intermediates. This makes secondary nucleation a harmful route, since oligomers are considered to be the neurotoxic species in the pathology of AD.^6−8^ The exact molecular mechanism of secondary nucleation and oligomer formation remains to be solved.

The coexistence of several Aβ sequence length variants with extensions and truncations at both termini has been found in vivo. In vitro studies have shown that extensions at the N-terminus reduce the rate constants of all microscopic steps, including secondary nucleation because the frequency of “productive” molecular collisions is reduced if the peptide is extended by a non-amyloidogenic segment.^4^ Indeed, the time at which half of the Aβ monomers are converted to fibrils grows exponentially with extension length and this dependence is replicated in Monte Carlo simulations of model peptides.^4^ In the fibrillar state, the unstructured regions surrounding the highly structured core may be termed the “fuzzy coat” and have been extensively studied for disease associated peptides such as α-synuclein and Tau.^9^

Several familial mutations at the N-terminal part of Aβ have been discovered to increase its aggregation propensity and neurotoxicity and lead to early onset Alzheimer’s disease.^10,11^ Of relevance for the current study, the A2V familial mutation increases the hydrophobicity of the N-terminal region and causes increased neurotoxicity and early onset AD.^12^ This mutation has only a small effect on the overall aggregation rate, but seems to increase the relative importance of secondary over primary nucleation.^13^ In contrast, the A2T familial mutation, which reduces the hydrophobicity, is reported to be protective against AD.^14^ Pyroglutamate at position three of Aβ is another disease-relevant N-terminal modification of Aβ42, which is shown to increase the neurotoxicity and oligomerization of Aβ.^15^ These findings imply a prominent role of the disordered N-terminal segment of Aβ in the nucleation and formation of neurotoxic oligomers, and in the pathology of Alzheimer’s disease.

One unresolved question regards whether secondary nucleation occurs on the sides of the ordered fibril core, or at the more disordered N-termini that decorate the fibril.^16^ High-resolution structures of the Aβ42 fibril formed in aqueous buffers at physiological pH have been reported,^17−19^ in which the N-terminal part of the peptide consisting of residues 1–14 is relatively unstructured. For Aβ42, the “fuzzy coat” flanking the highly ordered fibril core thus consists of the residues 1–14 at the N-terminal region. This part of the peptide contains three hydrophobic side-chains (A2, F4, and V12) and one of mixed hydrophobic/polar character (Y10). Most of the hydrophobic side-chains (L17, F19, F20, V24, A30, I31, I32, L34, M35, V36, V39, and I41) are buried in the fibril core, whereas the side-chains of residues V18, A21, V40, and A42 form two continuous hydrophobic patches along the surface of the fibril core.^17,18^ In a previous study, we investigated the role of these two hydrophobic patches in secondary nucleation.^20^ Secondary nucleation was found to be a dominating route of Aβ42 aggregation regardless of these hydrophobic residues; however, the replacement of some residues, most prominently V18, with polar ones led to the formation of an alternatively folded fibril structure that failed to catalyze the nucleation of monomers lacking this substitution.^20^ Other examples of failed cross-catalysis has been observed for Aβ40 versus Aβ42, and for all-L versus all-D Aβ20-34, in both cases related to different fibrillar structures of peptide variants.^21,22^ However, if the fibrils are totally different, formed from different proteins, some systems display an effect that can be explained as heterogeneous primary nucleation akin to the catalysis of primary nucleation on nanoparticle surfaces.^23^

**Figure 1:**
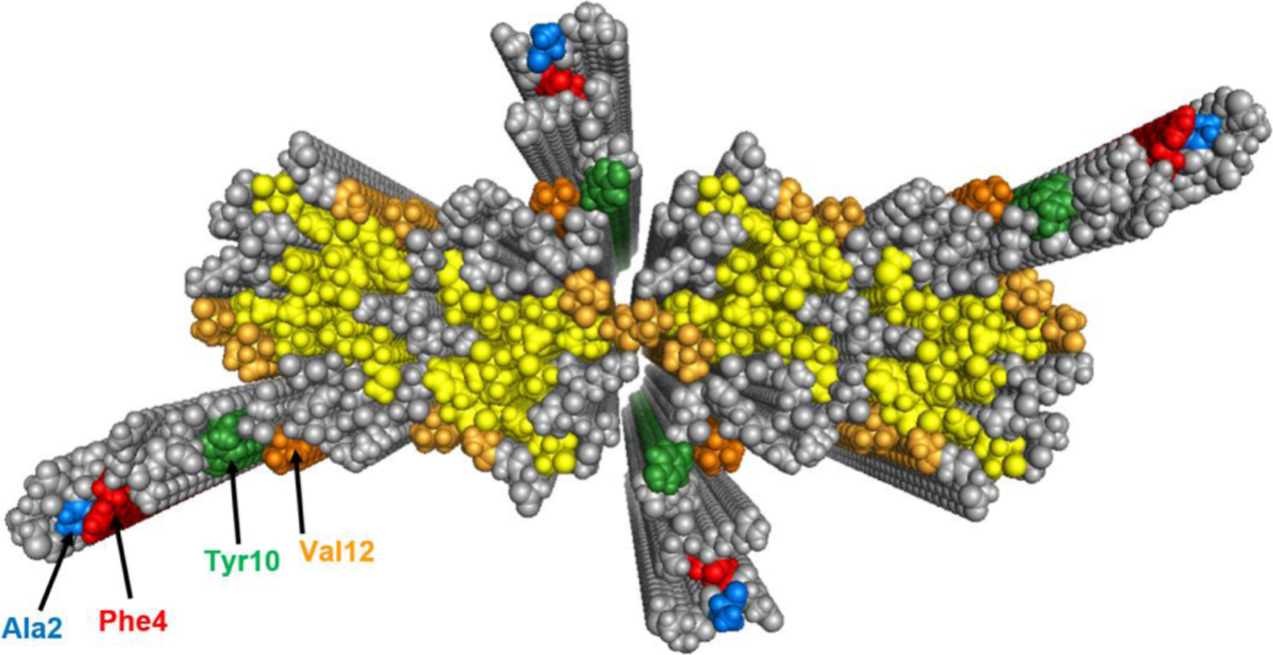
Structure of Aβ42 fibrils with four monomers per plane based on solid-state NMR^17^ and small angle X-ray scattering (SAXS),^19^ showing the hydrophobic residues buried in the fibril core (yellow) and the hydrophobic residues exposed on the surface of the fibril core (dark yellow). The residues investigated in this study are colored blue (A2), red (F4), green (Y10), and orange (V12).

In the present study, we investigate the role of the hydrophobic residues of the N-terminal region of Aβ42 in secondary nucleation by creating mutants with altered hydrophobicity or hydrophilic character. We ask whether modulation of the hydrophobicity at the N-terminal region alters the rate of secondary nucleation of the Aβ42 peptide, which in turn would affect the number of neurotoxic oligomers generated. We created nine single mutants A2T, A2S, F4A, F4S, Y10F, Y10A, Y10S, V12A, and V12S (Figure 1), and studied the concentration- and time-dependent aggregation kinetics using Thioflavin T fluorescence, while A2V has been studied in the same manner.^13^ To study the effect of hydrophobicity at the N-terminal region on surface-catalyzed nucleation, we performed self-seeding experiments of all mutants as well as cross-seeding with WT Aβ42. We also perform self- and cross-seeding studies for the mutant A2V with WT Aβ42. We used cryogenic Transmission Electron Microscopy (cryoTEM) to study the morphology of the mutant fibrils.

Several hydrophobicity scales have been developed over the last few decades, which take different factors into consideration to rank the amino acid residues according to their hydrophobicity. Certain scales are useful for predicting the transmembrane regions in proteins,^24,25^ while other scales are developed for identification of potentially antigenic sites in proteins.^26,27^ The early Wolfenden scale was based on the partitioning of amino acids between water and vapor phase,^28^ while a later one is based on the temperature effect on the hydrophobicity.^29^ Some scales reflect the average buried surface area of amino acids in globular proteins,^30,31^ and one scale ranks every amino acid relative to glycine.^32^ With the plethora of hydrophobicity scales available, some scales provide values based on a combination of other scales.^33,34^ The extent by which the current mutations alter the hydrophobicity is summarized over five such scales in Table 1. The substitutions of serine or alanine for aliphatic or phenyl residues are ranked as reducing the hydrophobicity by all these scales, except for Phe-to-Ala, which is ranked neutral by one of the five scales. Likewise Tyr-to-Phe is ranked as increasing the hydrophobicity by all scales. The assessments of the Tyr-to-Ser and especially Tyr-to-Ala substitutions are more variable.

**Table 1:** Effect of the Studied Mutations on the Hydrophobicity of the Peptide According to Different Hydrophobicity Scales^a^

mutant	Kyte-Doolittle	Cornette	Eisenberg	Monera	Wolfenden
A2S	***↓***	***↓***	***↓***	***↓***	*↓*
A2T	***↓***	***↓***	***↓***	***↓***	***↓***
F4A	***↓***	***↓***	***↓***	***↓***	***∼***
F4S	***↓***	***↓***	***↓***	***↓***	***↓***
V12A	***↓***	***↓***	***↓***	***↓***	***↓***
V12S	***↓***	***↓***	***↓***	***↓***	***↓***
Y10A	***↑***	***↓***	***↑***	***↓***	***↑***
Y10F	***↑***	***↑***	***↑***	***↑***	***↑***
Y10S	***↑***	***↓***	***↓***	***↓***	***↓***

a Downward arrows indicate reduced hydrophobicity, upward arrows increased hydrophobicity, tilde no change.

## Results

### Expression and Purification of Peptides

Sequence homogeneity and purity of the starting material are crucial for reproducible aggregation kinetics of peptides and its analysis. We thus expressed recombinant WT human Aβ42 as is, that is, without any tags except Met0, which is required to initiate the translation and purified from inclusion bodies using ion exchange and size exclusion steps, as described before.^35,5^ This mode of expression of Aβ(M1-42) peptides requires that the peptide has low enough solubility to form inclusion bodies, which avoids the degradation of small unstructured proteins in *Escherichia coli*. We found that all mutants of the current study could be expressed and purified to high homogeneity using the same protocol.

### Aggregation Kinetics

The fibril formation of the peptides was investigated under conditions at which Aβ42 is known to aggregate rapidly. Aggregation starting from freshly purified monomers was followed for samples of each peptide at a set of concentrations in the range of 1.1 to 10 μM by monitoring ThT fluorescence as a function of time at 37 ^*°*^C in 20 mM sodium phosphate, 0.2 mM ethylenediaminetetraacetic acid (EDTA), pH 8.0. Under these conditions, all mutant peptides form ThT-positive aggregates over time. The aggregation curves of the peptides A2T, A2S, F4A, F4S, Y10F, Y10A, V12A, and V12S have a sigmoidal-like appearance with a lag phase, a steep transition, and a final plateau, characteristic of nucleated polymerization reactions (see Figure 4). The Y10S mutant aggregates in a different manner compared to the other serine mutants, wherein at concentrations above 2.4 μM, the initial part of the transition is steep but the approach toward the final plateau is less distinct. We observe that for most of the mutants, the lag phase is extended and the overall aggregation retarded compared to WT Aβ42.

**Figure 2:**
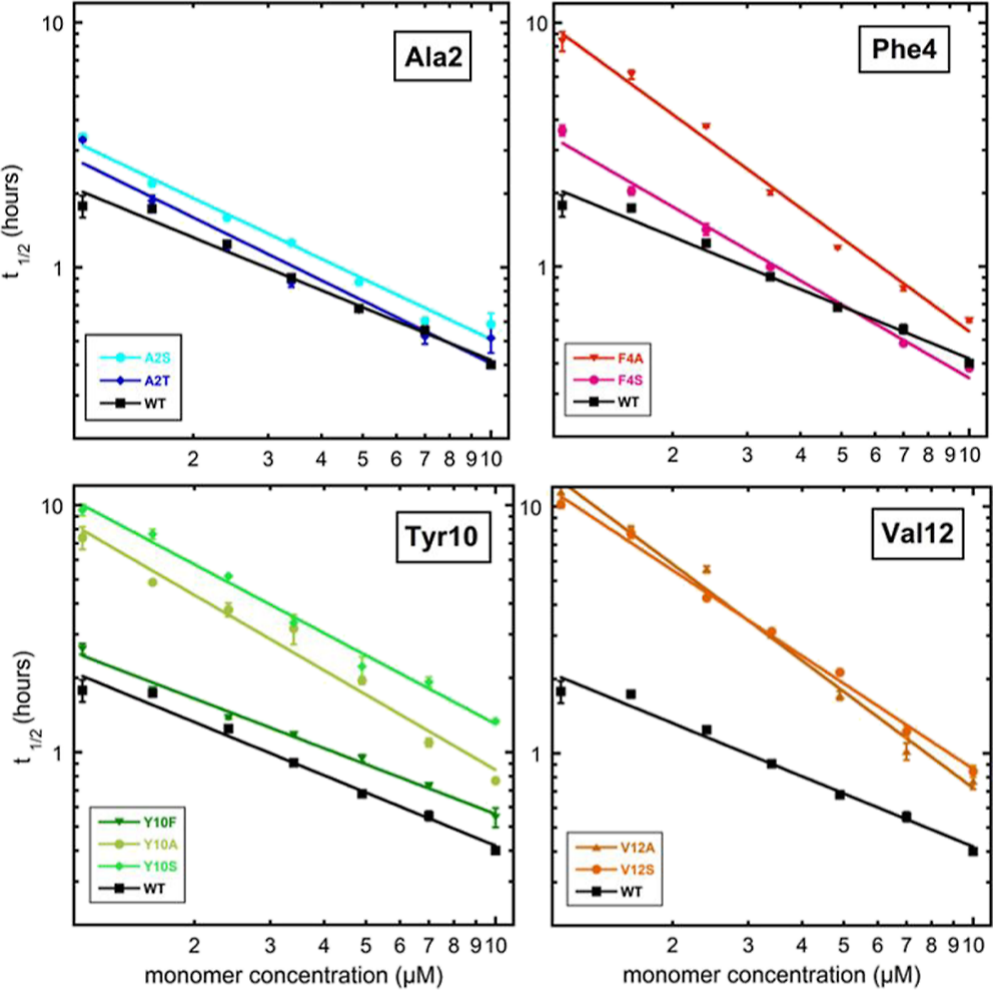
The time of half completion of the aggregation process, *t*_1/2_, is plotted on logarithmic scale as a function of peptide concentration for all mutants in comparison with WT Aβ42 in 20 mM sodium phosphate, 200 μM EDTA, 6 μM ThT, pH 8.0. Error bars represent the SD of three replicates, as shown in Figure 4, and the data from the three replicates are averaged.

The time at which half the monomer is converted to fibril, *t*_1/2_, versus the initial monomer concentration is shown in Figure 2 with logarithmic axes. We find that all mutants show retarded aggregation compared to the WT peptide over the entire concentration range, apart from the F4S mutant, which aggregates faster than WT at 10 μM. The mutants with aggregation behavior most similar to the WT peptide are A2T and F4S, with half-times t_1/2_ of 0.52 and 0.37 h, respectively, at 10 μM initial monomer concentration, and a concentration dependence similar to that of the WT. The mutants with reduced hydrophobicity at position 10 and 12 aggregate more slowly than WT, pointing to the importance of hydrophobicity at these positions. Y10S aggregates the slowest with a *t*_1/2_ of 1.32 h at 10 μM, compared to 0.40 h for the WT peptide. Y10A, V12A, and V12S also show a stronger dependence of t_1/2_ on peptide concentration compared to WT.

**Figure 3:**
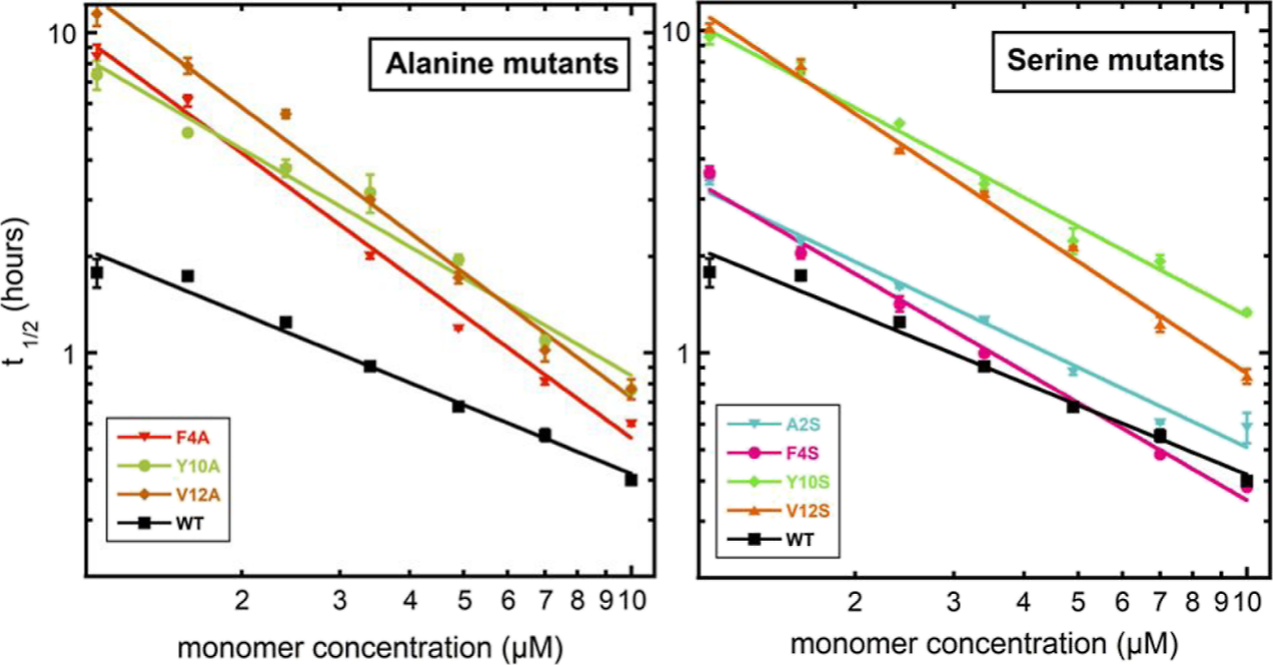
The time of half completion of the aggregation process, *t*_1/2_, is plotted on logarithmic scale as a function of peptide concentration for alanine and serine mutants in comparison to WT Aβ42 in 20 mM sodium phosphate, 200 μM EDTA, 6 μM ThT, pH 8.0. Error bars represent the SD of three replicates, as shown in Figure 4, and the data from the three replicates are averaged.

Figure 3 shows the comparative effect of introducing alanine or serine at all positions. Each alanine mutant aggregates more slowly than WT. Two serine mutants, Y10S and V12S aggregate more slowly than WT, while A2S and F4S display less effect. This indicates that the hydrophobicity at position 10 and 12 of Aβ42 is important for its self-assembly.

### Kinetic Analysis

**Figure 4:**
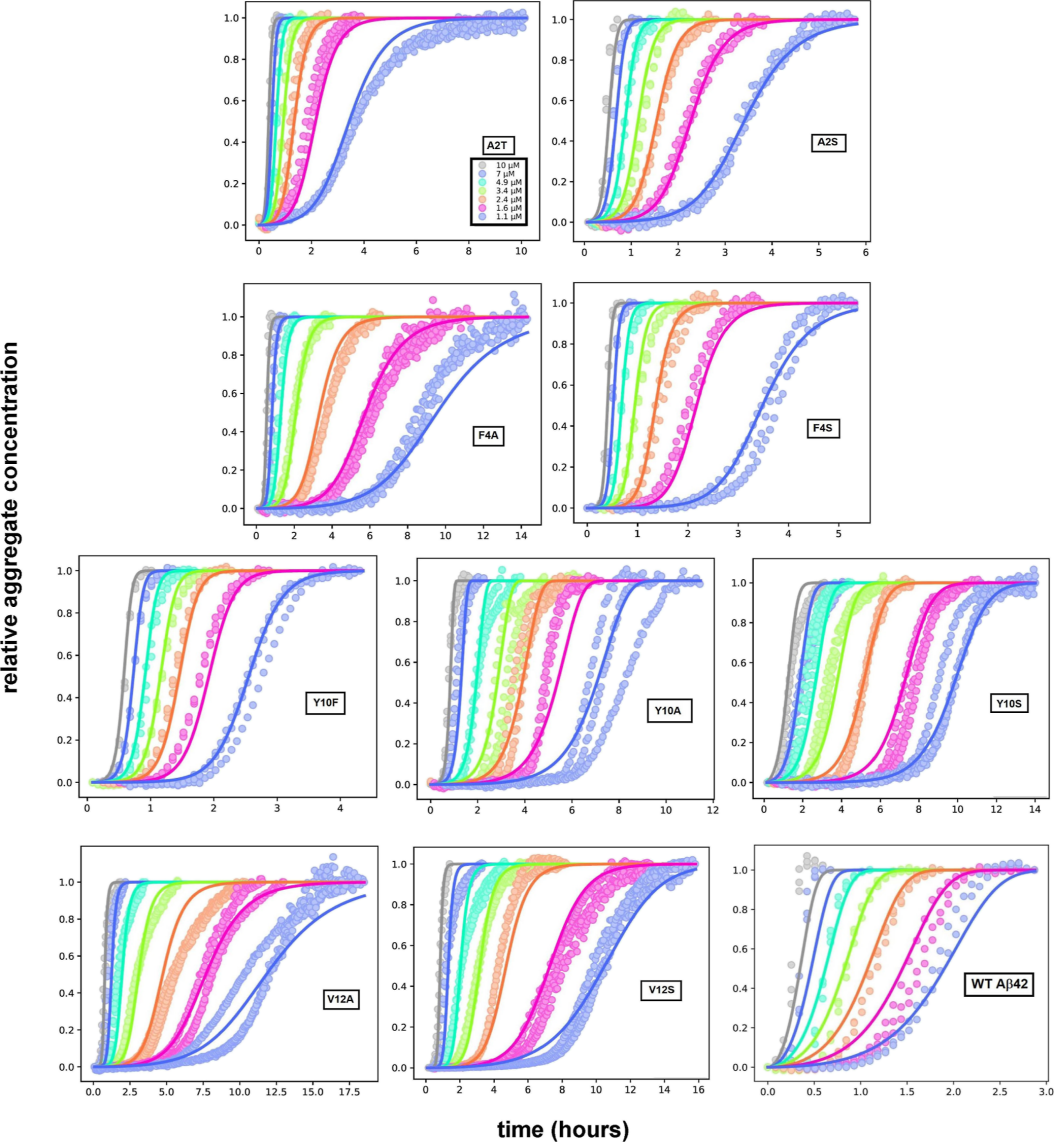
Aggregation kinetics for the nine mutant peptides as monitored by ThT fluorescence are shown in comparison with WT Aβ42. Aggregation was monitored in 20 mM sodium phosphate, 0.2 mM EDTA, 6 μM ThT, pH 8.0 for samples with 1.1, 1.6, 2.4, 3.4, 4.9, 7, and 10 μM of each peptide (color codes given in the top left panel are the same for all of the peptides). The data are examples showing one experiment with three replicates at each peptide concentration. The global fitting to all data for each peptide is shown with the curves at each concentration in the same color as the respective data points. The best fit was obtained using the multistep secondary nucleation dominant model for all peptides.

The aggregation data for each mutant were analyzed by global fitting of rate laws to the experimental data using the AmyloFit platform.^36^ This analysis connects macroscopic measurements of protein aggregation to the fundamental microscopic events, including nucleation and growth processes, which underlie the overall aggregation phenomenon, and to determine the microscopic rate constants of these processes. Through this approach, we can compare different peptide systems in terms of the molecular mechanism of aggregation. Models of varying complexity were tested, and we find that none of the data for the mutants can be fitted using models lacking secondary nucleation (see Supporting Information, Figure S1), whereas all data are well fitted by a model including secondary nucleation of monomers on the fibril surface (eq 1, Figure 4). This observation implies that secondary nucleation, which dominates the aggregation mechanism of the WT peptide, is retained as the key process by which new aggregates are formed for all mutants of the current study. Examples of aggregation data and the best fit using a model that includes multistep secondary nucleation are shown in Figure 4. This model describes an aggregation mechanism that consists of three microscopic steps, primary nucleation (rate constant *k*_n_), elongation (*k*_+_), and surface-catalyzed secondary nucleation (*k*_2_), and allows the catalytic surface for secondary nucleation to saturate, similar to the Michaelis–Menten model for enzyme kinetics.^37,38^ The kinetic analysis of unseeded aggregation experiments yields two effective kinetic parameters which are products of the microscopic rate constants, *k*_+_*k*_n_ and *k*_+_*k*_2_, referred to as the combined rate constants for the primary and secondary pathways, respectively, and a parameter, , describing the monomer concentration at half saturation of the secondary nucleation process (the reaction orders of the nucleation steps were assumed to be the same as in the WT). The measured aggregation kinetics initiated from the monomer state depend only on these products, not the rate constants individually.^39^ However, for the mutants displaying saturation in secondary nucleation, saturation is close to 100%, which means that *K*_M_ and *k*_+_*k*_2_ cannot be determined separately. For such cases, only the product of *k*_+_ and the maximal secondary nucleation rate *k*_2_*K*_M_ can be accurately reported, that is, *k*_+_*k*_2_*K*_M_.

**Figure 5:**
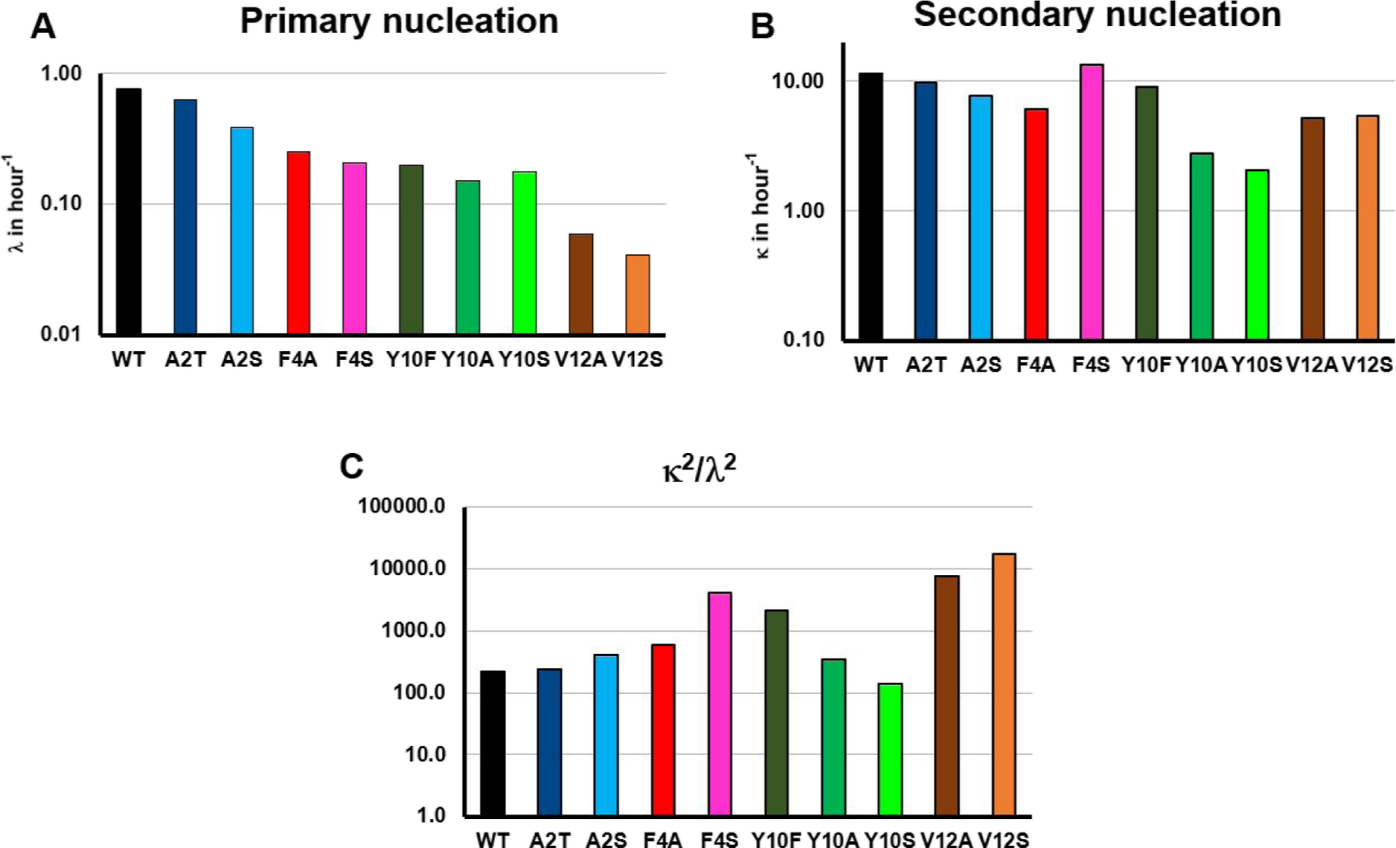
Decreasing N-terminal hydrophobicity reduces fibril formation rates of Aβ42, with hydrophobicity at position 10 being particularly important for secondary nucleation, and at position 12 for primary nucleation. Key fibril formation rate parameters are found from the global fitting of data for each Aβ42 mutant, evaluated at a representative monomer concentration of 5 μM, and plotted on a logarithmic scale. (A) Rate of proliferation of fibrils formed via primary nucleation. Large decreases are seen in all mutants except A2T and A2S, with V12A and V12S showing greater than tenfold decreases. (B) Rate of proliferation of fibrils formed via secondary nucleation. Large decreases are seen only for the mutants Y10A and Y10S, with all other mutants displaying little to no decrease. (C) The acceleration of nucleation by fibril surfaces, shown by κ^2^/λ^2^. Large increases are seen for the F4S, Y10F, and V12A/S mutants, due to large reductions in primary but not secondary nucleation rates. See the Discussion section for detailed structural and mechanistic interpretations of these changes.

Readily interpretable quantities, which can be used to compare the systems at different degrees of saturation, are the rates at which new fibril mass is formed through the pathways involving primary or secondary nucleation, denoted by λ and κ, respectively (see Materials and Methods for detailed definitions). These quantities, evaluated at a reference monomer concentration of 5 μM, are shown in Figure 5A,B, respectively. While it is meaningful to discuss changes of at least a factor of 2, the effects of the mutations on these quantities and other experimental observables are summarized in Table 2. The value of λ is decreased upon mutation at all studied positions, but to smallest extent for Ala2 mutants. The values of λ for the Phe4 and Tyr10 mutants are in the same range. The largest effect on primary processes is caused by mutation at position 12, with both V12A and V12S showing significantly lower λ. The value of λ for V12S is lowered by over one order of magnitude compared to WT. For the mutant F4S, κ is not significantly affected, but all other mutants show reduced rates of secondary processes. Reducing the hydrophobicity at positions 2 and 4 as in A2S and F4A lowers κ in comparison to WT. The most significant effect, however, is seen for Tyr10 and Val12 mutations. For Tyr10 mutations Y10S and Y10A, which alter the hydrophobicity at the 10th residue, κ is a factor of 3–5 lower than for WT. Additionally, for Val12 residue, lower hydrophobicity due to V12A and V12S causes κ to decrease approximately two-fold as compared to WT. Figure 5C shows the acceleration of nucleation by fibril surfaces in the form of the ratio κ^2^/λ^2^. Val12 mutants show an increased acceleration of nucleation by fibril surfaces despite the decreased κ. This can be attributed to the more significantly lowered rate of primary nucleation events. Y10S is the only mutant that has a slightly lower acceleration of nucleation by fibril surfaces as compared to WT. Even with the reduced κ, secondary nucleation is still prominent in all the mutants, as none of the data for the mutants can be fitted using mathematical models lacking secondary nucleation (Figure SI1).

**Table 2:** Columns are as follows. , where α_1_ is the rate of primary nucleation and α_2_ is the rate of secondary nucleation^a^

mutant	λ	κ	κ/λ	longer fibrils?	saturated?
A2S	***∼***	***∼***	***∼***	Y	Y
A2T	***∼***	***∼***	***∼***	Y	Y
F4A	***↓***	***∼***	***↑***	Y	N
F4S	***↓***	***∼***	***↑ ↑***	***∼***	Y
V12A	***↓ ↓***	***↓***	***↑ ↑***	Y	N
V12S	***↓ ↓***	***↓***	***↑ ↑***	Y	N
Y10A	***↓***	***↓***	***∼***	Y	N
Y10F	***↓***	***∼***	***↑ ↑***	***∼***	Y
Y10S	***↓***	***↓***	***∼***	Y	Y

a Fibril length trends are in the penultimate column, and mean fibril length is known to equal . The final column refers to the saturation of secondary nucleation. Double arrows mean changes of more than an order of magnitude. Tildes mean changes of less than a factor of 2.

### Morphology of Aggregates

**Figure 6:**
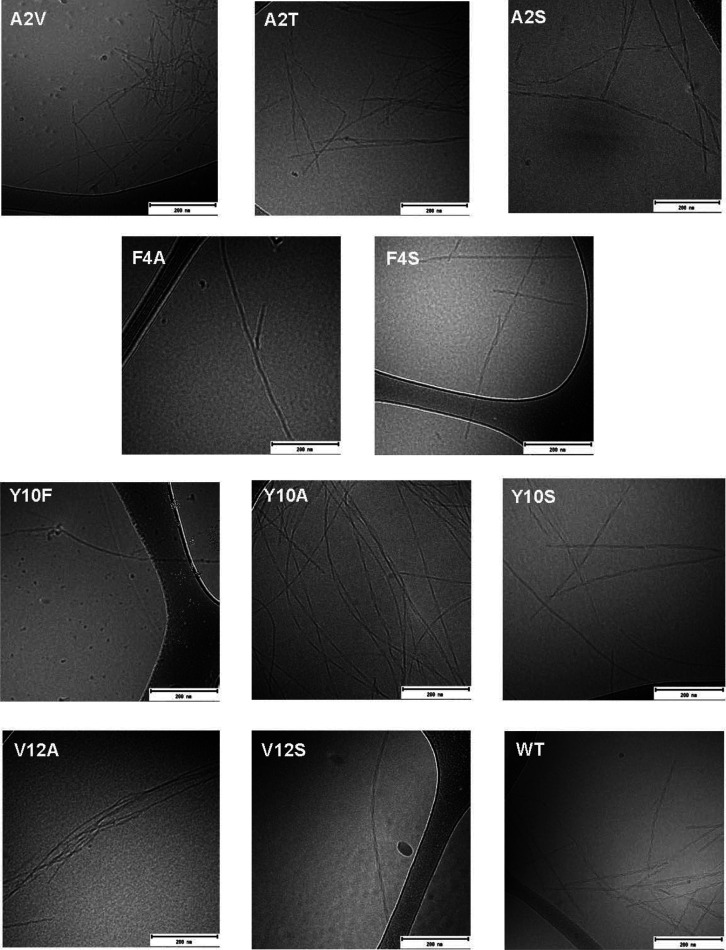
CryoTEM images of end-stage fibrils of the Aβ42 mutants A2V, A2T, A2S, F4A, F4S, Y10F, Y10A, Y10S, V12A, and V12S are shown in comparison with WT. A typical WT Aβ42 fibril shows the presence of two filaments twisted around each other in a way that creates nodes at regular intervals along the fibril.

CryoTEM was used to study the morphology of the end-stage fibrils for all mutants. In typical WT Aβ42 aggregates, individual filaments can be observed, and two filaments are twisted around each other along a common axis, seen as nodes that appear along the fibril at regular intervals (Figure 6). These fibrils are short and rigid and tend to cluster together on the sample grid, indicating highly hydrophobic behavior. This holds true also for the mutants with increased hydrophobicity compared to the WT peptide, such as A2V and Y10F. It is noteworthy that F4S shows the presence of a mixed population of fibrils, comprising of some very short fibrils as well as long fibrils. All the other mutants with lower hydrophobicity than the WT peptide seem to have only very long fibrils. This is the case for A2T, A2S, F4A, Y10A, Y10S, V12A, and V12S (Figure S2). The fibrils for all mutant peptides show node-to-node distances in the same range and similar to that of the WT peptide, indicating that all peptides form fibrils with similar structure as Aβ42 WT (Figure S3).

### Self-Seeding and Cross-Seeding Experiments

**Figure 7:**
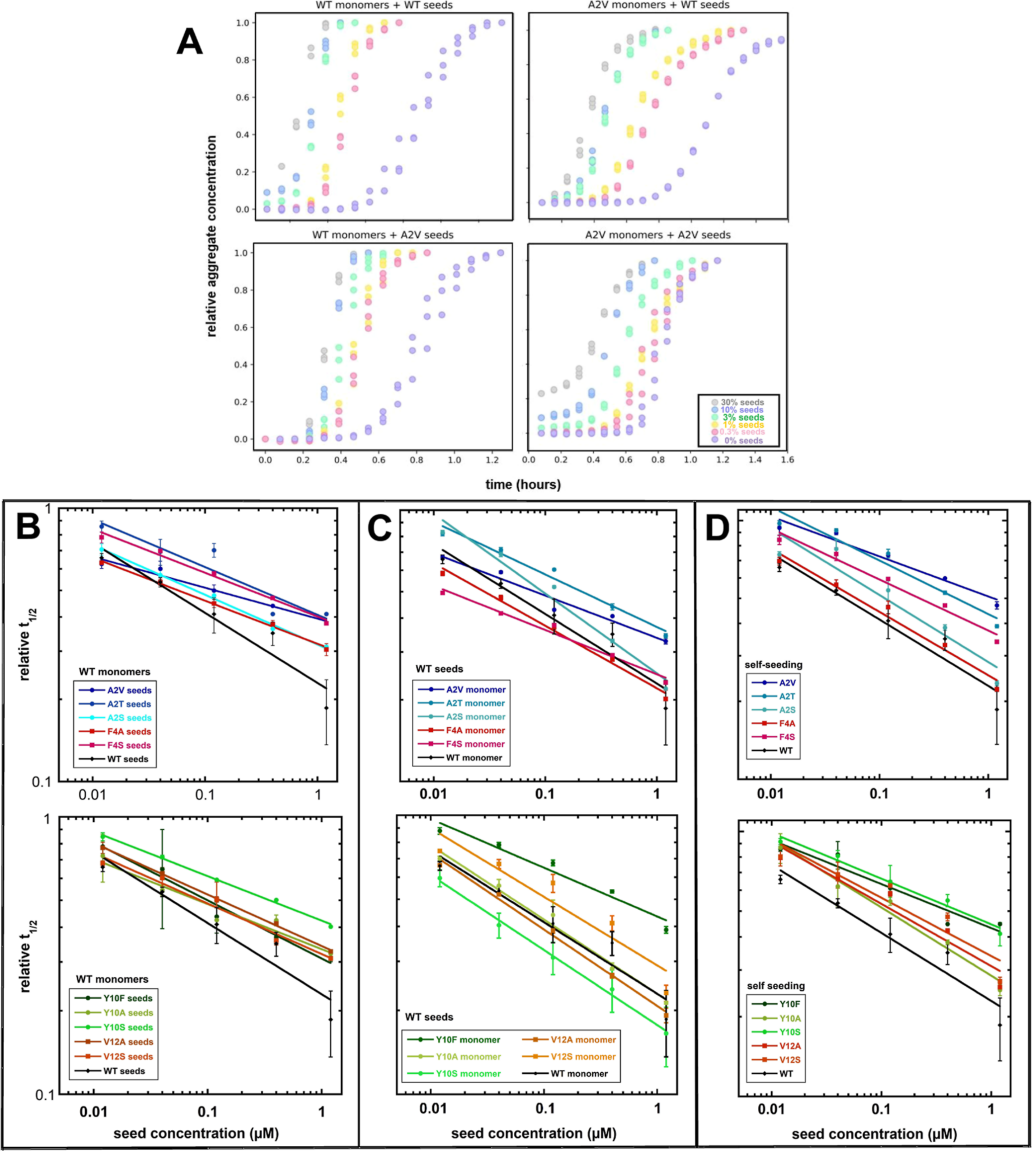
Self- and cross-seeding studies. (A) The self-seeding of WT Aβ42 and its cross-seeding with the A2V mutant is shown, as well as the self-seeding of A2V mutant and its cross-seeding with WT. (B) The time of half completion, *t*_1/2_, for cross-seeding experiments with monomers of WT Aβ42 on the seeds of each mutant is plotted on logarithmic scale as a function of seed concentration, shown in comparison with the self-seeding of WT. (C) The time of half completion, *t*_1/2_, for cross-seeding experiments with the monomers of each mutant on seeds of WT Aβ42 is plotted on logarithmic scale as a function of seed concentration, shown in comparison with the self-seeding of WT. Note: In the case of Y10S monomers on WT seeds, *t*_lag_ is plotted instead of *t*_1/2_, as the uncharacteristic aggregation curve shape makes it difficult to estimate *t*_1/2_. (D) The time of half completion, *t*_1/2_, for the self-seeding experiments of all mutants shown in comparison with the self-seeding of WT. All experiments are performed in 20 mM sodium phosphate and 0.2 mM EDTA at pH 8.0. Error bars represent the SD of three replicates as shown in Figure S4, and the data from the three replicates are averaged.

Self-seeding of each mutant was performed to validate the retained double-nucleation mechanism as inferred from the global kinetic analysis of non-seeded data. The self-seeding experiments were set up by adding preformed seeds of each mutant to freshly purified monomers of the same mutant. Cross-seeding of each mutant with WT Aβ42 was performed in order to probe whether the WT seeds catalyze the nucleation of the mutant monomers and vice versa. The cross-seeding experiments were set up by adding preformed seeds of WT Aβ42 to freshly purified monomers of each mutant, and in converse by adding preformed seeds of each mutant Aβ42 to freshly purified monomers of the WT Aβ42. In all these experiments, the seed concentrations ranged from 0.3 to 30% of the monomer concentration in monomer units in steps of a factor of three. We also performed seeded aggregation kinetics for the mutant A2V, for which unseeded and self-seeded aggregation was previously studied.^13^ The results are shown in Figure S4 and summarized in Figure 7.

The lag phase is expected to decrease as the seed concentration increases if self- or cross-catalysis is effective. The results thus imply that substitutions at position 2, 4, 10, and 12 do not hinder cross-catalysis. The nucleation of WT monomers is catalyzed equally well with seeds of A2V, A2T, and A2S mutants as they are with WT seeds. Likewise, the nucleation of monomers of all three Ala2 mutants is catalyzed strongly by WT seeds. The same result is found for F4A, F4S, Y10A, Y10F, V12A or V12S versus WT. None of these substitutions alter significantly the nucleation of WT monomers on mutant seed or vice versa.

The results show that for all mutants, both self-seeding and cross-seeding with WT are highly effective in shortening the lag phase. The imposed changes in surface character are thus not critical for surface-catalyzed nucleation.

## Discussion

The current study was motivated by the early-onset AD observed for individuals carrying the A2V mutation^12^ or the pyro-glutamate-3 modification^15^ and by the protective role reported for the A2T substitution.^14^ These findings point to a significant role of the hydrophobicity of the N-terminal region of Aβ42, which was here investigated in a systematic manner through the mutagenesis of all four hydrophobic residues in this region, A2, F4, Y10, and V12. Our analysis and interpretation rely on a significant amount of progress made over the last few years in terms of understanding the microscopic steps involved in the aggregation mechanism of Aβ42. These microscopic steps include primary nucleation, elongation, and secondary nucleation. During secondary nucleation, fibrils provide a catalytic surface for the conversion of monomers into larger aggregates, thus causing a proliferation in the amount of fibril mass produced. The surface properties of fibrils may thus have an effect on the catalytic behavior of the fibril. Aggregation studies of familial and designed mutants provide clues to the molecular driving forces of nucleation processes. For example, the net negative charge of fibrils and monomers seem to limit the nucleation rate through electrostatic repulsion, a factor which is alleviated in several familial mutants with less negative charge.^40^ The same reasoning holds in opposite direction; designed mutants that make the net charge of Aβ40 more negative, and the peptide more hydrophilic, have been found to significantly reduce the aggregation propensity.^41^ In a previous study, we investigated the role of hydrophobic surfaces exposed on the fibril surface of Aβ42 on secondary nucleation.^20^

The introduced point mutations at Ala2, Phe4, Tyr10, and Val12 of Aβ42 create mutant peptides with more hydrophilic or larger or smaller hydrophobic side chains at these positions as compared to WT (see Table 1). The results of non-seeded and seeded aggregation kinetics reveal that all mutants aggregate with the same mechanism as WT, albeit with saturation of secondary nucleation for the five mutants A2S, A2T, F4S, Y10F, and Y10S. Such saturation of secondary nucleation is not observed for WT, F4A, V12A, V12S, and Y10A in the peptide concentration range studied here.

The individual and combined rate constants as obtained from global fitting to the data (Figure 5, Table 2) reveal that for all mutants the largest effect is on the rate constant for primary nucleation. The less hydrophobic peptides are less prone to hydrophobic collapse and thereby less prone to nucleate in solution. The reduced hydrophobicity is also expected to lead to increased solubility of the peptides, an equilibrium property. An extreme case is provided by the P3 peptide, that is, Aβ17-42 with the entire N-terminal region missing.^42,43^ The P3 peptide is characterized by reduced solubility and accelerated nucleation.

While the rate constant for secondary nucleation is retained or reduced for all mutants with reduced hydrophobicity, the decrease in secondary nucleation rate, parameter κ, never exceeds and is often smaller than the reduction of primary nucleation rate, parameter λ. This effect is consistent with the findings from a previous study, where serine substitutions in place of the hydrophobic residues exposed on the Aβ42 fibril surface led to the resulting mutants displaying a κ at least one order of magnitude higher than λ.^20^ This implies the mutations do not selectively affect secondary nucleation and the overall effects of reduced hydrophobicity are larger than those arising from changes in the fibril surface properties. Indeed, the cross-seeding data suggest retained catalytic efficiency of fibrils of all mutants, and cryoTEM images suggest a similar fibril fold for all variants.

Serine substitutions at positions 2, 4, 10, and 12 were created by the mutations A2S, F4S, Y10S, and V12S. The F4S mutation does not cause significant changes in the secondary nucleation rate compared to WT. However, secondary nucleation for F4S becomes saturated, suggesting alterations to individual steps within the secondary nucleation process, such as arrival, conversion, and detachment, whose effects broadly cancel one another out (Table 2). The analysis indicates that fibrils get stickier and that conversion and detachment of fibril-bound monomers to form new oligomers/fibrils is slowed down. The Sabatier principle states that there is an optimal substrate affinity above which reduced product release impedes catalysis and below which catalysis is reduced due to insufficient substrate binding.^44^ A2S, and in particular Y10S and V12S, show a decrease in fibril mass formed via secondary nucleation. Y10S and V12S also show an extended half time of aggregation, t_1/2_ and a significantly reduced primary nucleation rate. The Y10S mutation conserves the polar −OH group while reducing the hydrophobicity at position 10 in Aβ42. The F4S and Y10S mutations both result in the loss of an aromatic ring, causing both a decrease in hydrophobicity and in the size of the side chain at the respective position. However, only Y10S shows a significant reduction of secondary nucleation, pointing to the position dependence in the role of amino acid side chains in this process.

Alanine substitutions at positions 4, 10, and 12 (mutants F4A, Y10A, and V12A) lead to a decrease in κ, with the most significant effect for Y10A, which also shows an extended lag phase of aggregation. The V12A mutation involves a smaller decrease in hydrophobicity, with the loss of one −CH_3_ and one −CH_2_ group at position 12, while F4A involves a larger loss in hydrophobicity and side chain size. The larger reduction of κ found for Y10A, points to position-dependent roles. At position 10, we also studied the mutant Y10F, which removes the polar nature of Tyr10 but maintains the hydrophobic and aromatic character. Y10F has only a slightly lowered secondary nucleation rate compared to WT, showing that the −OH group of Tyr10 is not crucial for secondary nucleation.

A previous study involving the familial AD mutant A2V showed that A2V mutation causes an increase in secondary nucleation rate as compared to WT Aβ42.^13^ A2V mutation is pathogenic and involves an increase in hydrophobicity through the addition of one −CH_3_ and one −CH_2_ group. Another familial mutant, A2T, is protective in nature,^14^ with a side chain of mixed hydrophobic/polar nature at position 2. This pointed to a role of the hydrophobic group of Ala2 in the neurotoxicity and thus secondary nucleation of Aβ42. To test this, we created A2S, which preserves the polar character, but removes the hydrophobic −CH_3_ group, to test whether the lack of hydrophobicity altered the secondary nucleation of Aβ42. However, both A2T and A2S display similar rate of secondary nucleation as compared to WT.

Since for all variants, secondary nucleation dominates over primary nucleation, the main factor governing the fibril length distribution is the relative rates of secondary nucleation and elongation. The cryoTEM imaging data reveal similar fibril morphology of all mutants (Figure 6, Figure S2), while F4A, V12A, V12S, Y10A, and Y10S form fibrils which appear significantly longer than WT fibrils. This may reflect a larger decrease in secondary nucleation relative to elongation. F4S shows the coexistence of long and very short fibrils, in line with its increased rate of secondary nucleation. Mutants with increased hydrophobicity, namely, A2V and Y10F, also form shorter fibrils, indicative of increased rate of secondary nucleation relative to elongation.

Previous findings have shown that the surface-catalyzed nucleation is dependent on structural compatibility between the nucleating monomers and the fibril structure.^20^ We therefore studied the self-seeded aggregation kinetics for all mutants in this study, as well as their cross-seeding with WT Aβ42. Aggregation of WT monomers is found to be strongly accelerated by WT as well as all mutant seeds, leading to a significant shortening the lag phase in a seed-concentration dependent manner. Likewise, the aggregation of each mutant peptide is accelerated by mutant seeds as well as WT seeds. The results show that for all mutants, both self-seeding and cross-seeding with WT are highly effective. The imposed changes in surface character are thus not critical for surface-catalyzed nucleation and there appears to be structural compatibility between the fibrils of each mutant and WT monomers as well as between WT fibrils and mutant monomers. The results imply that the secondary nucleation is a robust feature retained by all mutants of the current study.

### Concluding Remarks

The results of this study clearly show that the hydrophobicity of the N-terminal region of Aβ42 is a contributing factor to the relatively high rates of primary and secondary nucleation of this peptide. Reduced hydrophobicity at the N-terminal region of Aβ42 leads to a strong retardation of primary nucleation. Comparing the four positions with hydrophobic side-chains in the WT Aβ42 N-terminal region, this effect is the most prominent for the Val12 residue. Additionally, the hydrophobic residues, particularly Tyr10 and Val12, play an important role in the secondary nucleation of Aβ42. This can be seen in terms of the fibril mass produced via secondary nucleation being lowered for the mutants with smaller hydrophobic groups at these positions. Most of the mutants with reduced hydrophobicity at the N-terminal region form longer fibrils as compared to WT Aβ42, pointing to the reduced rate of consumption of monomers via secondary nucleation. These elongated fibrils still show similar fibril morphology, which can be characterized by the node-to-node distances of the fibrils. These findings highlight that while hydrophobicity of the N-terminal region of Aβ42 is important for secondary nucleation, it is not the only factor governing secondary nucleation, which still remains a dominant process in the aggregation process. Indeed, because primary nucleation is most strongly affected, secondary nucleation is even more dominant in the double-nucleation mechanism of all mutants compared to WT.

Based on the results of the current and previous studies, we can make the following summary regarding the driving forces for Aβ42 self assembly. The net negative charge of the peptide counteracts fibril formation and limits the nucleation rate. Salt screening or change mutations therefore lowers the peptide solubility and increases the rates of primary and secondary nucleation, the latter to the extent that it saturates at high peptide concentration. The main driving force for Aβ42 self assembly is the hydrophobic effect. Clearly the nucleation rate is governed not only by those hydrophobic residues that become buried in the hydrophobic core of the fibril but also by those that end up on the surface of the core and in the unstructured N-terminal region. Thus, the low solubility of Aβ42 due to the high prevalence of hydrophobic residues promotes nucleation through a general hydrophobic effect and the tendency of the monomers to come out of aqueous solution.

## Materials and Methods

### Expression and Purification of Peptides

The plasmid carrying synthetic genes with *E. coli-*optimized codons for Aβ42 WT (PetSac, cloned by us^35^) as well as A2V, A2T, A2S, F4A, F4S, Y10F, Y10A, Y10S, V12A, and V12S (Pet3a, purchased from Genscript) were transformed into Ca^2+^ competent cells of *E. coli* strain BL21 DE3 pLysS star and the protein was expressed in auto-induction medium.^45^ The peptides were purified using ion exchange chromatography (IEX) as described^35^ with the minor change that lower salt concentration (50 mM NaCl) was used to elute the peptides, and size exclusion chromatography (SEC) on a 26 × 600 mm Superdex 75 column was used instead of spin filters for molecular mass fractionation. The purified monomeric peptides were lyophilized as aliquots until further use.

### Preparation of Samples for Kinetic Experiments

The lyophilized aliquots of the purified peptides were dissolved in 1 mL of 6 M GuHCl, 20 mM sodium phosphate, 0.2 mM EDTA, pH 8.5, and subjected to gel filtration on a Superdex 75 10/300 column in 20 mM sodium phosphate buffer pH 8.0, with 0.2 mM EDTA. EDTA was included to control any metal ion concentrations to very low. The middle part of monomer peak was collected in a low-binding tube (Axygen) on ice, and was typically found to have a concentration in the range 20–80 μM (determined by absorbance of the collected part of the chromatogram peak using ϵ_280_ = 1400 L mol^–1^ cm^–1^). The collected monomer was supplemented with 6 μM thioflavin T (ThT) from a 2.7 mM stock, and dilutions were performed as explained below.

### Aggregation Kinetics by Thioflavin T Fluorescence

Aggregation kinetics experiments were performed as a function of peptide concentration. The highest concentration of the peptide was 10 μM, and the solution was logarithmically diluted with the buffer to final concentrations ranging down to 1.1 μM into a 96-well plate (Corning 3881), 100 μL per well using a tailor-made pipetting robot.^46^ The experiments were initiated by placing the 96-well plate at 37 ^*o*^C in a plate reader (Fluostar Omega). The ThT fluorescence was measured through the bottom of the plate every 120 s using an excitation filter at 440 nm, and an emission filter at 480 nm.

Self-seeding and cross-seeding experiments were performed using a constant monomer concentration of 4 μM. The seed concentrations were 30, 10, 3, 1, 0.3, and 0% in monomer equivalents. A monomer stock solution of 8 μM concentration was prepared in a low binding tube. Seeds were prepared from a starting monomer concentration of 10 μM in a 96-well plate. These were then diluted to 2x the final concentrations. All dilutions were performed in 20 mM sodium phosphate buffer pH 8.0, with 0.2 mM EDTA and 6 μM Thioflavin T. 50 μL of the desired seeds were added from the respective 2x stock solutions, and then 50 μL of the monomer solution was added to each well from the stock, giving a total of 100 μL of 1x seeds + 1x monomers in each well.

### Analysis of Aggregation Kinetics

The analysis of the aggregation kinetics to determine the molecular mechanism and the rate constants underpinning this process was performed using the fitting platform AmyloFit,^36^ at which the kinetic data were uploaded, normalized, and fitted. This analysis uses equations derived by considering the contributions from primary nucleation, secondary nucleation, and elongation. The model for a multistep secondary nucleation dominated process was successful in fitting to all the experimental data. In this model, the fraction of aggregated proteins at time *t* is given by
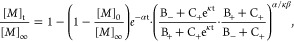
1where the definitions of the parameters are
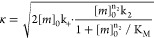

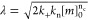

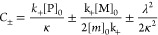

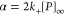

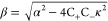

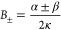
and where [*m*]_0_ is the initial monomer concentration; [*P*]_0_, [*M*]_0_, and [*P*]_∞_, [*M*]_∞_ are the aggregate number and mass concentration at the beginning and end of the aggregation, respectively (see ref (36) for detailed expression of [*P*]_∞_); k_+_, k_*n*_, and k_2_ are the rate constants of elongation, primary, and secondary nucleation, respectively; and *n*_*c*_ and *n*_2_ are the reaction orders of primary and secondary nucleation, respectively.

### CryoTEM

For all peptides, samples of 10 μM monomer were incubated at 37 ^*°*^C in PEGylated plates (Corning 3881) in a plate reader and collected after reaching the plateau in ThT fluorescence. Specimens for cryoTEM were prepared in an automatic plunge freezer system (Leica EM GP). The climate chamber temperature was kept at 21 ^*°*^C, and relative humidity was 90% to minimize the loss of solution during sample preparation. The specimens were prepared by placing 4 μL solution on glow discharged lacey formvar carbon-coated copper grids (Ted Pella) and blotted with filter paper before being plunged into liquid ethane at −183 ^*°*^C. This leads to vitrified specimens, avoiding component segmentation and rearrangement, and the formation of water crystals, thereby preserving original microstructures. The vitrified specimens were stored under liquid nitrogen until measured. A Fischione model 2550 cryo transfer tomography holder was used to transfer the specimen into the electron microscope, JEM 2200FS, equipped with an in-column energy filter (Omega filter), which allows zero-loss imaging. The acceleration voltage was 200kV, and zero-loss images were recorded digitally with a TVIPS F416 camera using SerialEM under low dose conditions with a 10 eV energy selecting slit in place.

## Supporting Information Available

Kinetic analysis, cryogenic electron micrographs, analysis of fibril morphology, and self- and cross-seeding experiments (PDF)
