# Supplementary material for: Role of Hydrophobicity at the N-Terminal Region of Aβ42 in Secondary Nucleation
Source: ACS Chem Neurosci. 2022 Nov 21;13(23):3477–87. doi: 10.1021/acschemneuro.2c00504 (PMC9732875; doi:10.1021/acschemneuro.2c00504)
Supplement: Supplementary file 1 — cn2c00504_si_001.pdf [file cn2c00504_si_001.pdf]

Supporting information

Dev Thacker,[†] Amanda Willas,[†] Alexander J. Dear,[†,‡] and Sara Linse[*,†]

†*Department of Biochemistry and Structural Biology, Lund University, Sweden 22362.*

‡*Centre for Misfolding Diseases, Department of Chemistry, University of Cambridge, Cambridge CB2 1EW.*

E-mail: sara.linse@biochemistry.lu.se

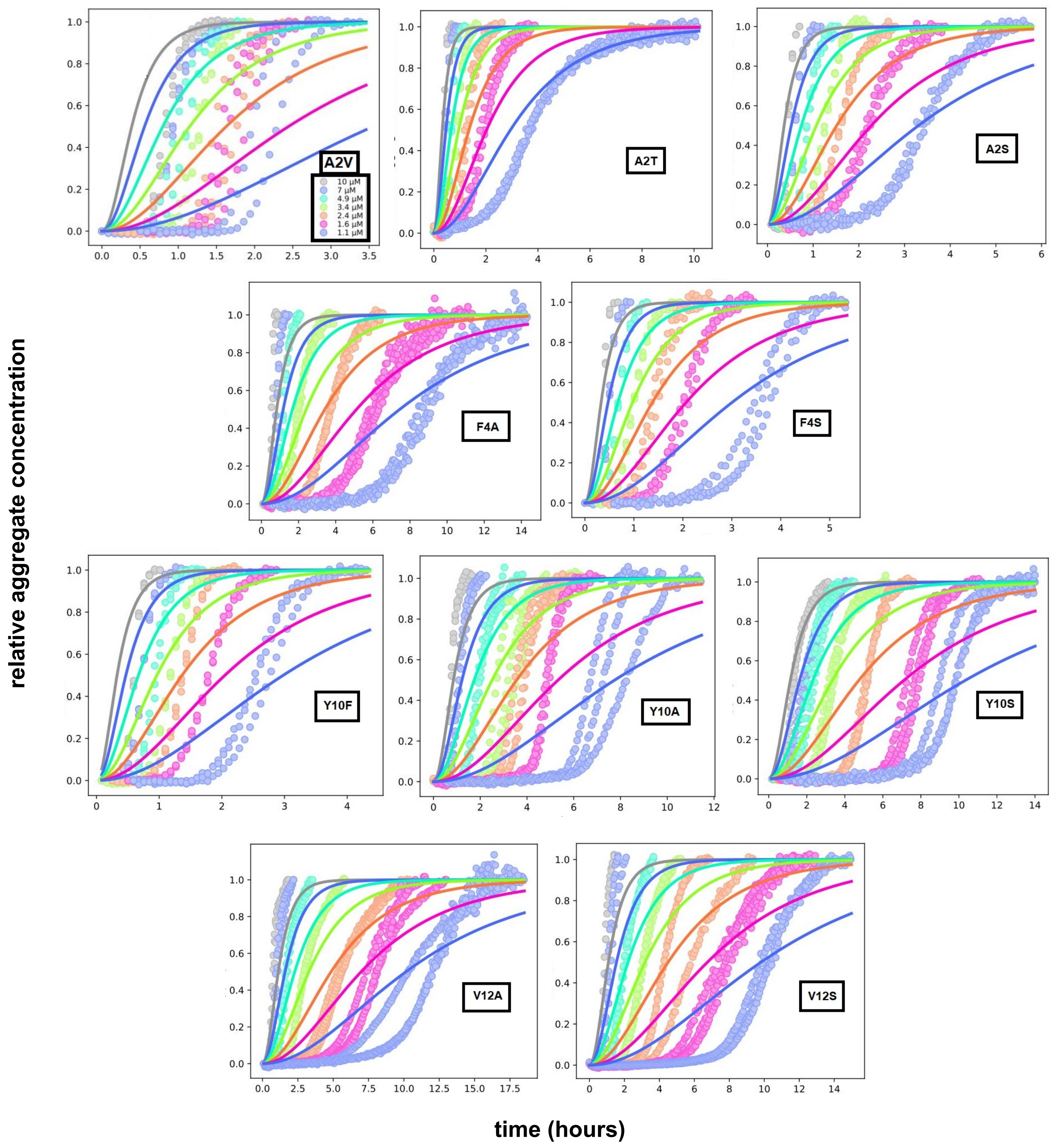

Figure S1: Examples of a bad fit with a model which lacks secondary nucleation (nucleation elongation model) are shown here for the experimental data of all the mutants from figure 4.

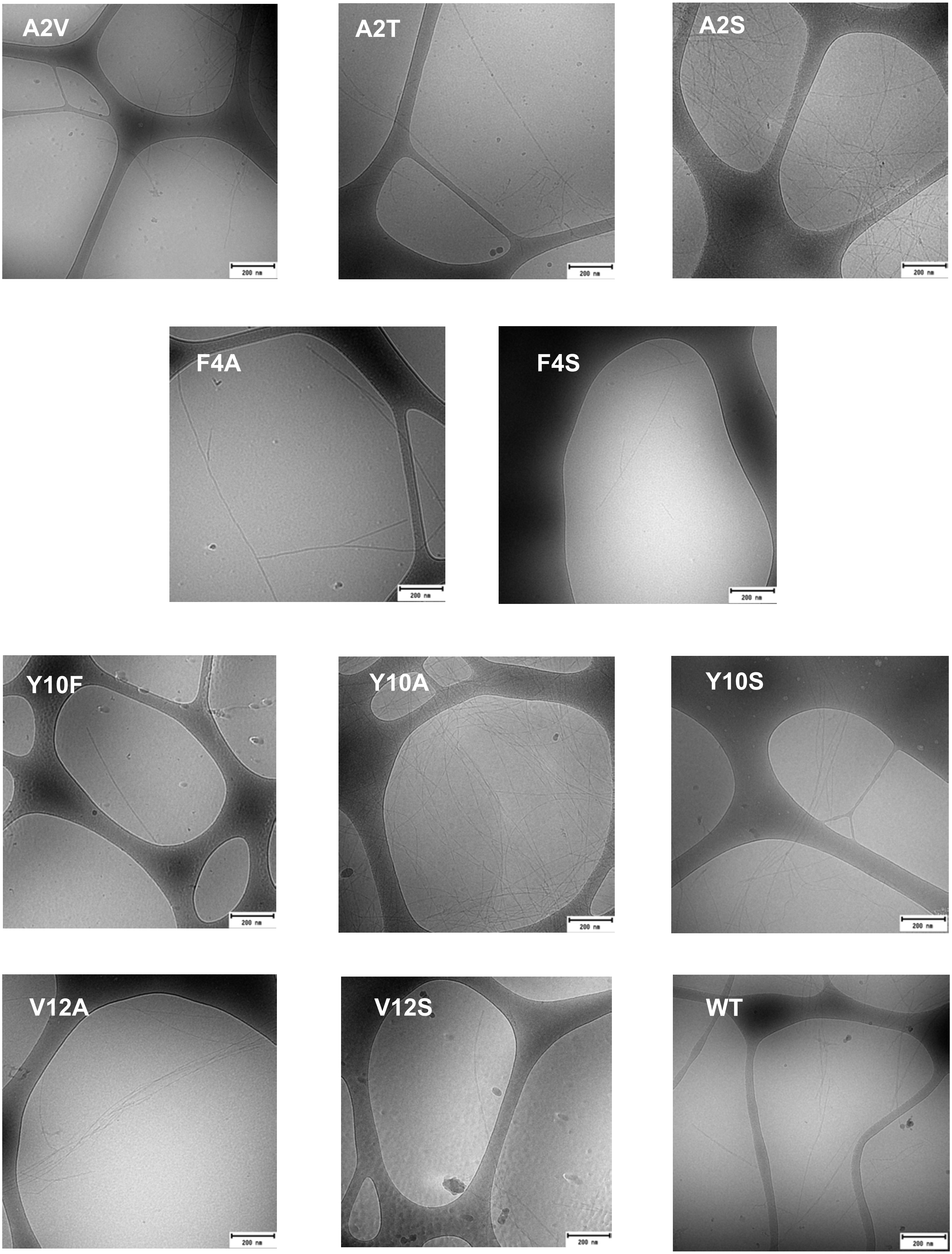
A2V
200 nm
A2T
200 nm
A2S
200 nm
F4A
200 nm
F4S
200 nm
Y10F
200 nm
Y10A
200 nm
Y10S
200 nm
V12A
200 nm
V12S
200 nm
WT
200 nm

Figure S2: CryoTEM images of end-stage fibrils of the mutants A2V, A2T, A2S, F4A, F4S,Y10F, Y10A, Y10S, V12A, and V12S are shown in comparison with WT. A typical WTAβ42 fibril shows the presence of two filaments twisted around each other in a way that creates nodes at regular intervals along the fibril. Fibrils formed by the mutants show similar morphology to WT Aβ4 2 fibrils, however fibrils of mutants with lowered hydrophobicity are markedly longer than WT, pointing to larger monomer utilization via elongation.

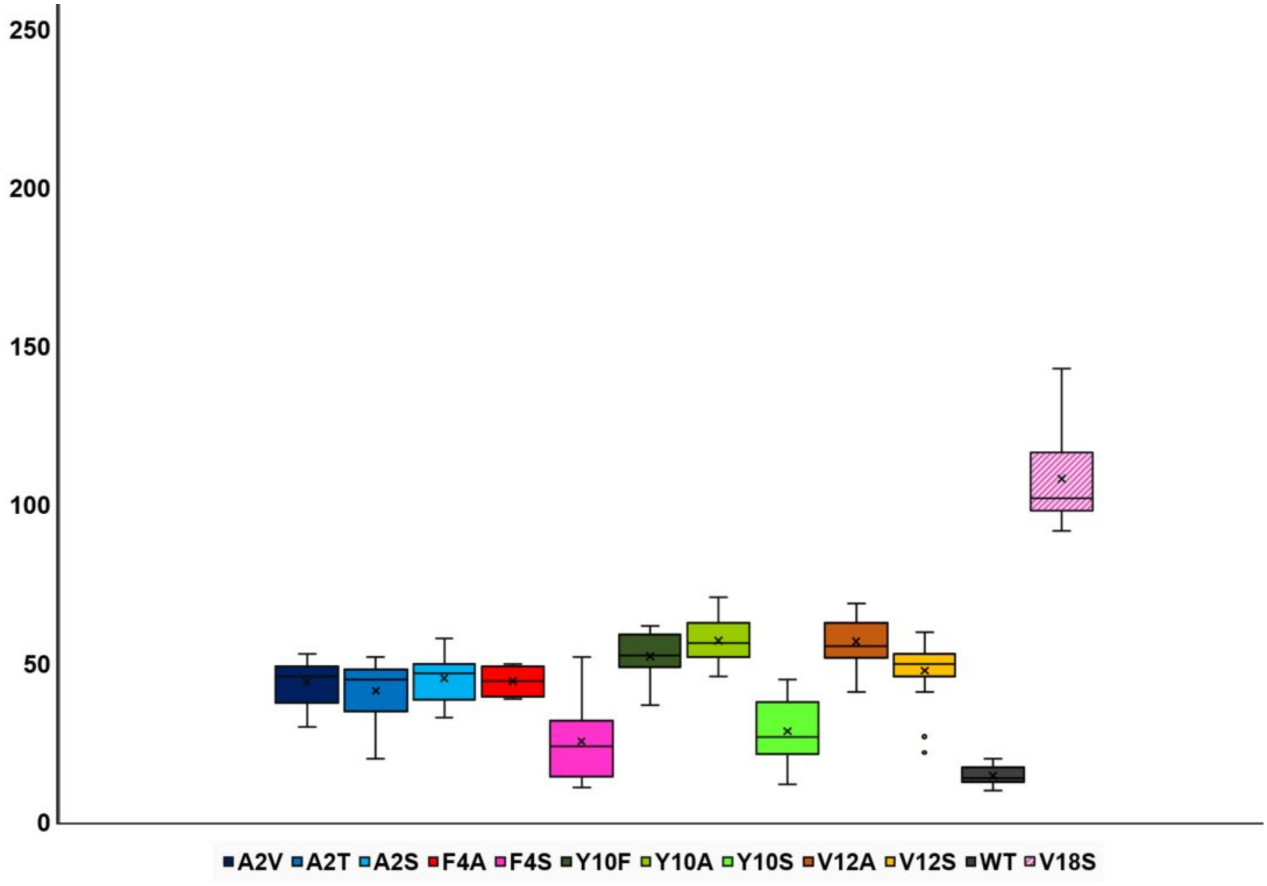

Figure S3: Analysis of the fibril morphology based on the cryoTEM images (figure 6) in terms of node-to-node distance distributions. Node-to-node distance for a fibril can be defined as the distance between two consecutive nodes created by the twisting of the two filaments of a fibril on top of each other at regular intervals. This analysis shows that all mutants show similar fibril morphology as WT Aβ42. An example of a mutant V18S Aβ42 which forms fibrils of a different morphology is shown for comparison from a previous study.

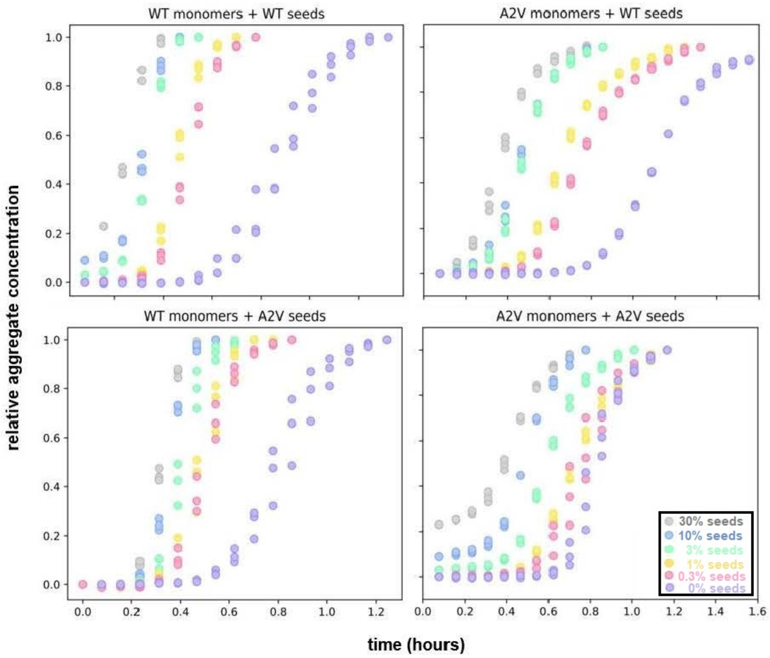
WT monomers + WT seeds
A2V monomers + WT seeds
WT monomers + A2V seeds
A2V monomers + A2V seeds
relative aggregate concentration
time (hours)
30% seeds
10% seeds
3% seeds
1% seeds
0.3% seeds
0% seeds

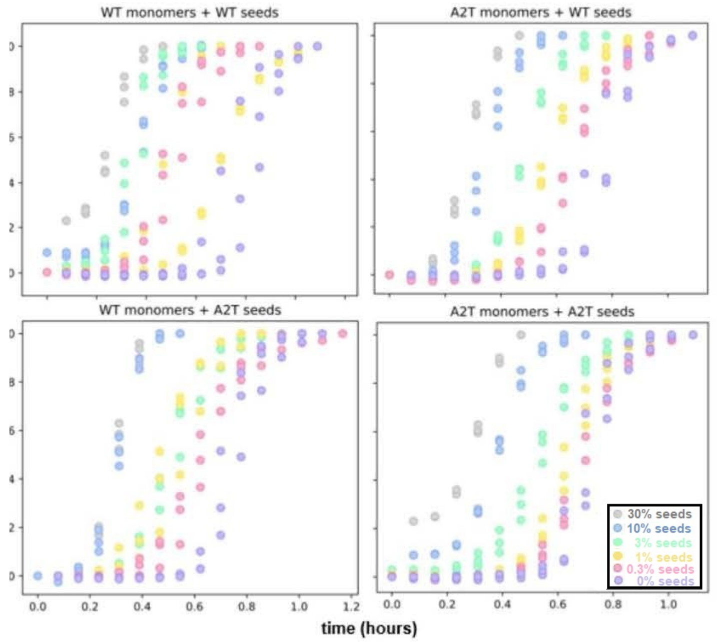
WT monomers + WT seeds
A2T monomers + WT seeds
WT monomers + A2T seeds
A2T monomers + A2T seeds
time (hours)
30% seeds
10% seeds
3% seeds
1% seeds
0.3% seeds
0% seeds

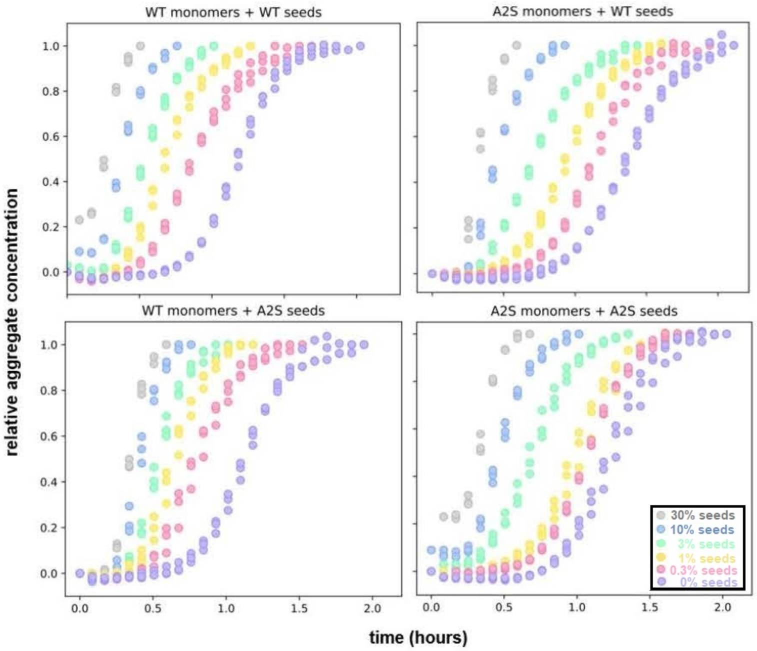
WT monomers + WT seeds
A2S monomers + WT seeds
WT monomers + A2S seeds
A2S monomers + A2S seeds
relative aggregate concentration
time (hours)
30% seeds
10% seeds
3% seeds
1% seeds
0.3% seeds
0% seeds

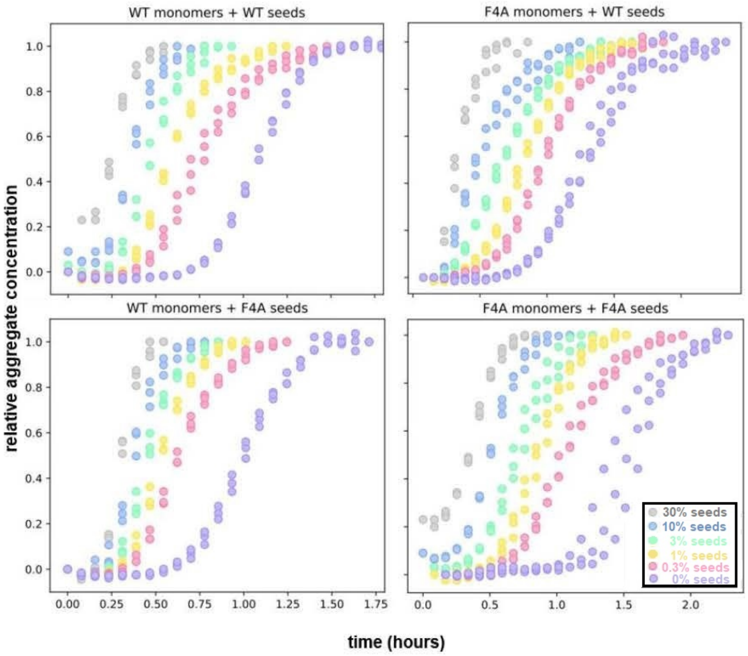
WT monomers + WT seeds
F4A monomers + WT seeds
WT monomers + F4A seeds
F4A monomers + F4A seeds
relative aggregate concentration
time (hours)
30% seeds
10% seeds
3% seeds
1% seeds
0.3% seeds
0% seeds

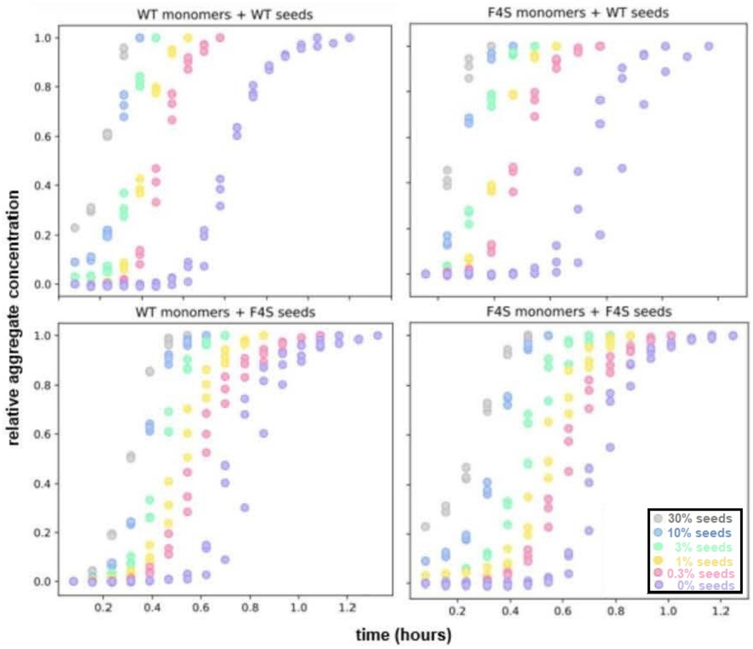
WT monomers + WT seeds
F4S monomers + WT seeds
WT monomers + F4S seeds
F4S monomers + F4S seeds
relative aggregate concentration
time (hours)
30% seeds
10% seeds
3% seeds
1% seeds
0.3% seeds
0% seeds

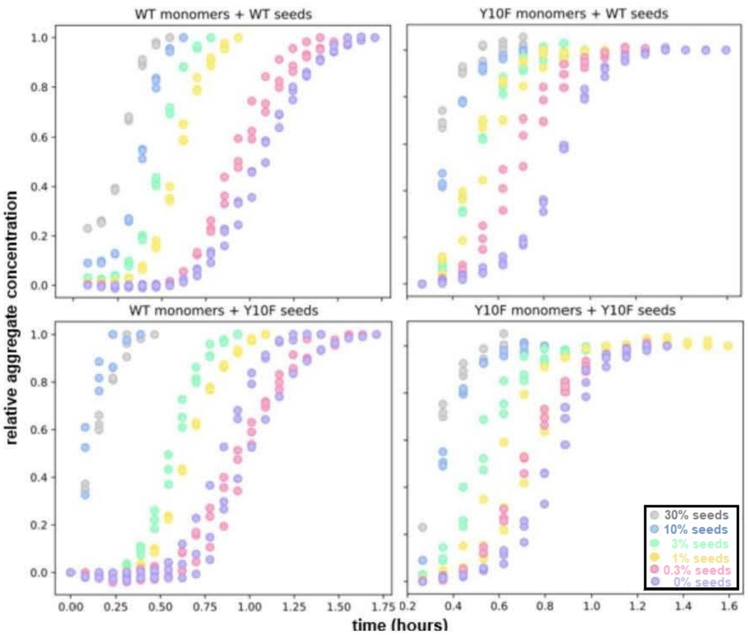
WT monomers + WT seeds
Y10F monomers + WT seeds
WT monomers + Y10F seeds
Y10F monomers + Y10F seeds
relative aggregate concentration
time (hours)
30% seeds
10% seeds
3% seeds
1% seeds
0.3% seeds
0% seeds

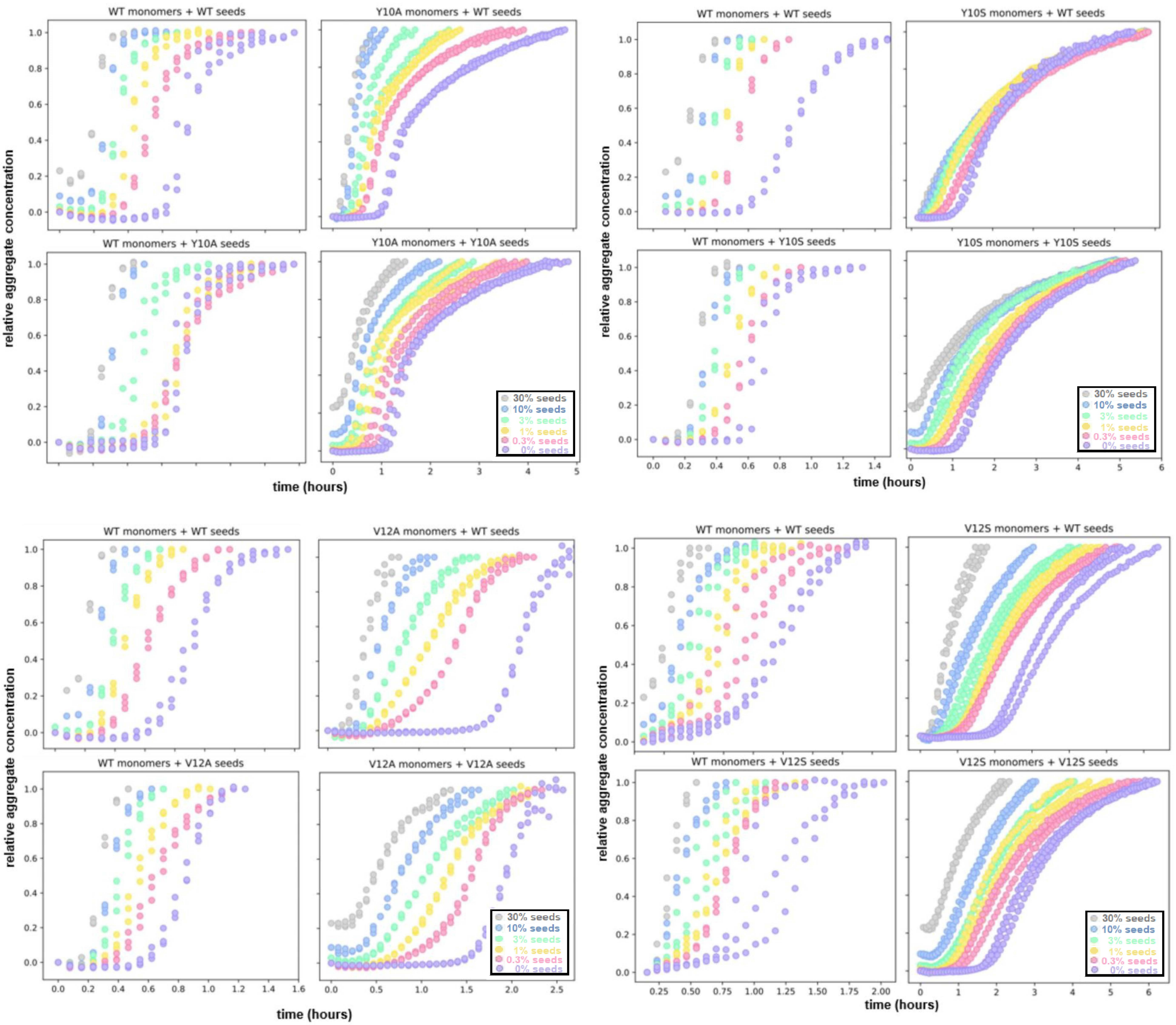

Figure S4: Self- and cross-seeding experiments of WT Aβ42 and all mutants in presence of 6 μM ThT in 20 mM sodium phosphate, 200 μM EDTA at pH 8.0. For both self- and cross-seeding kinetics, the monomer concentration was 4 μM. Seed concentrations were 30% (grey), 10% (blue), 3% (green), 1% (yellow), 0.3% (pink), and 0% (purple) in monomer equivalents.
